# Editorial: Current Challenges for Targeting Brown Fat Thermogenesis to Combat Obesity

**DOI:** 10.3389/fendo.2020.600341

**Published:** 2020-10-27

**Authors:** Takeshi Yoneshiro, Rosalía Rodríguez-Rodríguez, Matthias Johannes Betz, Patrick C. N. Rensen

**Affiliations:** ^1^Division of Metabolic Medicine, Research Center for Advanced Science and Technology, The University of Tokyo, Tokyo, Japan; ^2^Diabetes Center, University of California, San Francisco, San Francisco, CA, United States; ^3^Basic Sciences Department, Faculty of Medicine and Health Sciences, Universitat Internacional de Catalunya, Barcelona, Spain; ^4^Department of Endocrinology, Diabetes and Metabolism, University Hospital Basel and University of Basel, Basel, Switzerland; ^5^Division of Endocrinology, Department of Medicine, Leiden University Medical Center, Leiden, Netherlands; ^6^Einthoven Laboratory for Experimental Vascular Medicine, Leiden University Medical Center, Leiden, Netherlands

**Keywords:** brown adipose tissue, beige adipocytes, energy metabolism, thermogenesis, obesity, type 2 diabetes

## Introduction

Just over a decade has passed since metabolically active brown adipose tissue (BAT) was unequivocally identified in healthy adult humans. Since then, researchers have provided evidence of expression of the thermogenic molecule uncoupling protein 1 (UCP1) in human BAT, as well as its energy dissipating capacity. Additionally, clinical cross-sectional analysis suggested that a decline in BAT activity with aging, as judged from [^18^F]FDG-PET/CT, coincides with the development of obesity and insulin resistance. These major observations provided a rationale to investigate BAT as a potential target for preventing obesity. Indeed, several studies have demonstrated that cold exposure and adrenomimetic agents strongly activate BAT thermogenesis, and thus energy expenditure in humans. However, the extent to which stimulating BAT thermogenesis decreases adiposity in humans remains unclear. Moreover, therapeutic strategies aimed at general stimulation of the sympathetic nervous system (SNS) and adrenergic receptors involved may not be applicable for obese and diabetic patients, because of potential negative side-effects on cardiovascular function.

While implementation in humans remains challenging, recent studies in mice have yielded insights in molecular mechanisms underlying thermogenic regulation of brown and brown-like (brite/beige) adipocytes, highlighting their ability to reduce insulin resistance and atherosclerosis (1). These include identification of UCP1-independent thermogenesis as well as peripheral organ-derived endocrine factors and metabolites capable of triggering adipocyte thermogenesis. Activating these non-canonical mechanisms through genetic manipulation or pharmacological agents suppresses obesity and its associated complications in mice. The major challenge remains translating preclinical scientific advances to humans. To bridge basic research with clinical investigation, this Research Topic reviews the current advances in human BAT pathophysiology, novel molecular mechanisms regulating adipose thermogenesis, and remaining challenges in this field.

## Physiology and Pathology of BAT

Numerous studies have confirmed the contribution of BAT to cold-induced thermogenesis (CIT) in mice and humans. Additionally, diet-induced thermogenesis (DIT), another component of non-shivering thermogenesis, has demonstrated reliance on BAT in mice—evidence in humans is limited. The review article by Saito et al. gathers the recent advancements in human BAT physiology, focusing on the contribution of BAT to DIT. The authors provide an overview of the mechanisms by which food intake triggers BAT thermogenesis, including the SNS, bile acids, secretin, and ghrelin.

Another physiological stimulus of thermogenesis in BAT and white adipose tissue (WAT) is exercise. Vidal and Stanford provide an overview of the molecular mechanisms underlying exercise-induced adipose thermogenesis. The authors also discuss how exercise-induced release of lipokine 12,13-diHOME from BAT can stimulate fatty acid metabolism in skeletal muscle. Notably, environmental factors, such as ambient temperature, mediate the effects of exercise on adipose thermogenesis. Aldiss et al. demonstrate that in rats, exercise does not induce browning at thermoneutrality but instead induces a muscle-like signature in BAT, which might be relevant to the previously uncharacterized UCP1-independent thermogenesis in BAT. Because of the divergent results between rodents and humans, further studies in both species are needed to fully understand the physiological meaning, molecular mechanisms, and clinical significance of adipose tissue adaptation in response to exercise.

In addition to exercise, intriguingly, certain disease states have also been shown to mediate BAT activity. Increased BAT activity in patients with cancer is a known aspect of cachexia-induced body-weight loss. Conversely, BAT thermogenesis may possibly be impaired in patients with a different disease, narcolepsy type 1, caused by the destruction of orexin-producing neurons in the hypothalamus (Straat et al.). Orexin-producing neurons control the wake-sleep cycle and appetite, but also participate in SNS-mediated BAT activation. Although patients with narcolepsy type 1 exhibit increased adiposity, convincing evidence in humans demonstrating impaired BAT function with narcolepsy type 1 is still lacking.

Chronic inflammation in adipose tissue poses a significant metabolic challenge, as it impairs BAT thermogenesis and exacerbates insulin resistance. Omran and Christian comprehensively review the effect of inflammatory cells, such as macrophages and mast cells, on the function of thermogenic adipocytes. The activity of inflammatory cells is mediated by proinflammatory cytokines, generating an inflammatory microenvironment.

## Methodological Advancements

[^18^F]FDG-PET/CT has emerged as the gold standard for assessing the metabolic activity of BAT in humans. However, given the challenges in performing repeated [^18^F]FDG-PET/CT including exposure to ionizing radiation, methodological advances to estimate BAT activity are highly desirable.

Wu et al. extensively describe the currently available modalities to non-invasively probe BAT volume and/or metabolic activity, especially focusing on magnetic resonance imaging (MRI) and spectroscopy (MRS) methods. MR techniques have enabled assessment of the lipid content, microstructure, and the mitochondrial oxidative capacity of BAT. The authors also discuss the inherent limitations in quantifying BAT by MR. To this end, Abreu-Vieira et al. demonstrate the fundamental importance of adjusting fat fraction thresholds to accurately determine cold-induced changes in BAT *via* MR. Another novel methodology for estimating BAT in humans is near-infrared time-resolved spectroscopy (NIR_TRS_), which measures absolute concentration of hemoglobin (Hamaoka et al.). As BAT is highly vascularized, NIR_TRS_ parameters are indicative of BAT volume/activity.

In addition to the aforementioned imaging techniques, BAT functionality might be estimated from the circulating BAT-derived secretome (i.e. “batokines”) or microRNAs, although the utility of these metrics is debated. Similarly, BAT-associated metabolites may well be indicative of BAT activity. In this regard, oxygenated polyunsaturated fatty acid metabolites (oxylipins), could be a surrogate marker for BAT activity and/or amount, as Dieckmann et al. show that murine BAT is distinguishable from WAT by oxylipin profiling.

These advances in non-invasive methodology are potentially valuable in performing future cross-sectional studies with large numbers of participants as well as longitudinal studies with repeated BAT measurements, thereby facilitating investigations into BAT biology in humans.

## New Thermogenic Mechanisms

The identification of novel molecular circuits involved in BAT thermogenesis may lead to the discovery of targetable pathways for the development of practicable pharmacotherapies for adiposity and related cardiometabolic diseases. Recent studies in small rodents have contributed to substantial advances in this regard. While thermogenesis in brown adipocytes largely relies on UCP1 function, brite/beige adipocytes residing in WAT can utilize alternative pathways that are independent of UCP1. Ikeda and Yamada review the current evidence of UCP1-independent thermogenic pathways involved in creatine-substrate cycling and calcium cycling. It remains essential to determine the contribution of non-canonical thermogenesis to whole-body energy homeostasis in humans.

The β-adrenergic receptor (β-AR) is the crucial gatekeeper of adipose thermogenesis. Several studies have unveiled additional signaling pathways mediated by adenosine and mineralocorticoid receptors as reviewed by Pan et al. Subsequently, they introduce β-AR-independent mechanisms by which cold exposure induces a unique type of thermogenic adipocyte with enhanced glucose oxidative capacity, so-called “glycolytic beige”, contributing to systemic glucose homeostasis in mice.

New insights also include novel transcription factors and related molecules involved in adipose thermogenesis. Müller et al. identify the orphan nuclear factor estrogen related receptor gamma (ESPRG), and the transcriptional factor PGC1 and ESRR-induced regulator in muscle 1 (PERM1) as modulators of cold-induced browning of WAT. They show ESPRG and PERM1 positively regulate UCP1 expression and mitochondrial components, respectively.

## Metabolites Regulate Thermogenesis

The SNS is undoubtedly a central regulator of adipose thermogenesis. A concept gaining increasing attention is that non-neuronal systems can also trigger adipose thermogenesis. Indeed, peripheral organ-derived endocrine factors and metabolites can play a role in adipose thermogenesis through transcriptional control and non-genomic post-translational modification. In this context, Herz and Kiefer describe that intracellular retinoids stringently control browning of WAT through the regulation of *Ucp1* transcription and protein retinoylation. Retinoid action can be stimulated by norepinephrine, implying the importance of retinoids in cold-induced adipose thermogenesis.

Moreover, the review by Bast-Habersbrunner et al. provides an in-depth overview of intracellular mechanisms by which cytosolic purine nucleotides control UCP1 function. Purine nucleotides such as ATP, ADP, GTP, and GDP have been proven to be constitutive inhibitors of UCP1-mediated proton conductance. Adrenergic stimulation dramatically reduces cytosolic purine nucleotides, facilitating UCP1-mediated thermogenesis. Further elucidation of the signaling cascade of the adrenergic receptor-mediated control of cytosolic purine nucleotides may help establish approaches to directly modulate BAT thermogenesis.

## Perspectives

It is clear that thermogenic adipocytes and their precursors, once thought monolithic, are composed of various, distinct cell populations (2, 3). For instance, although conventional brite/beige adipocytes arise from MyoD^-^ progenitors, the glycolytic beige adipocytes arise from MyoD^+^ progenitors (Pan et al.). Due to the complex heterogeneity, the developmental origin and pathways remain insufficiently understood. A better understanding of this heterogeneity may lead to the development of new approaches to specifically activate certain subpopulations of thermogenic cells without unfavorable side-effects.

In addition, mechanisms governing the generation of precursors committed to thermogenic adipocyte cell fate are still poorly understood. Given the difficulty of obtaining an abundant source of human progenitors for study, pluripotent stem cells (PSCs), as implemented by Yao et al., are promising tools to elucidate such mechanisms. However, several challenges have been presented, including the weak efficacy of thermogenic differentiation, which precludes greater utilization. Another future challenge will be the generation of efficient *in vitro* models resembling human adipose tissue, in which an efficient interplay between the various cell types involved is likely important.

From a clinical perspective, further studies to translate the proofs of concept generated in rodent models to clinical investigation are crucial. Although there are many similarities between rodent and human BAT depots, notable differences also exist. Whereas the activation of murine BAT is mediated by β3-AR, a recent study revealed that β2-AR is the relevant adrenoceptor which activates human BAT (4). This underscores the need for caution when translating new advances generated by preclinical studies to humans. Future research should focus on the cardiometabolic benefits of long-term activation of β2-AR, preferably BAT-specific, in obese patients.

Lastly, further methodological improvements in visualization and evaluation of BAT metabolic activity in humans are warranted to define the actual contribution to whole-body nutrient turnover and energy expenditure. Of note, given that mouse studies suggest the uptake by BAT of triglyceride-derived fatty acids may be higher than that of glucose, and lipoprotein lipase is crucial for murine BAT thermogenesis, development of a PET-compatible lipoprotein-triglyceride tracer may be valuable to accurately estimate the total energy dissipating capacity of human BAT.

In conclusion, the original and review articles published in this Research Topic have provided significant insights in the field of thermogenic fat biology, emphasizing new aspects of pathophysiology and molecular control of BAT thermogenesis both in preclinical and clinical settings. We also emphasize the need for further investigation of the physiological significance of human BAT, methodological advances in estimating BAT functionality, novel non-canonical thermogenic pathways, and the heterogeneity of thermogenic adipocytes. Pursuing these issues may open opportunities to develop effective strategies utilizing adipose tissue thermogenesis to combat obesity and cardiometabolic diseases in humans.

## Author Contributions

TY wrote the manuscript. RR-R, MB, and PCNR edited the manuscript. All authors contributed to the article and approved the submitted version.

## Funding

TY is supported by the research fellowship from the Uehara Memorial Foundation. RR-R is supported by the Spanish Ministry of Economy, Industry and Competitiveness (SAF2017-82813-C3-3R) and Joint Bilateral Project Japan-Spain/Agencia Estatal de Investigación (PCI2018-092997/AEI). MB is supported by the Swiss National Science Foundation (PZ00P3_167823). PCNR is supported by the Netherlands CardioVascular Research Initiative (CVON-GENIUS-II).

## Conflict of Interest

The authors declare that the research was conducted in the absence of any commercial or financial relationships that could be construed as a potential conflict of interest.
